# Effects of a Therapeutic Exercise Protocol for Patients with Chronic Non-Specific Back Pain in Primary Health Care: A Single-Group Retrospective Cohort Study

**DOI:** 10.3390/jcm12206478

**Published:** 2023-10-12

**Authors:** Juan Nicolás Cuenca-Zaldívar, Josué Fernández-Carnero, Eleuterio A. Sánchez-Romero, Verónica Álvarez-Gonzalo, Rocío Conde-Rodríguez, David Rodríguez-Sanz, César Calvo-Lobo

**Affiliations:** 1 Grupo de Investigación en Fisioterapia y Dolor, Departamento de Enfermería y Fisioterapia, Facultad de Medicina y Ciencias de la Salud, Universidad de Alcalá, 28801 Alcalá de Henares, Spain; nicolas.cuenca@salud.madrid.org; 2 Research Group in Nursing and Health Care, Puerta de Hierro Health Research Institute-Segovia de Arana (IDIPHISA), 28222 Majadahonda, Spain; 3 Interdisciplinary Research Group on Musculoskeletal Disorders, Faculty of Sport Sciences, Universidad Europea de Madrid, 28670 Villaviciosa de Odón, Spain; 4 Department of Physical Therapy, Occupational Therapy, Rehabilitation and Physical Medicine, Rey Juan Carlos University, 28032 Madrid, Spain; 5 Grupo Multidisciplinar de Investigación y Tratamiento del Dolor, Grupo de Excelencia Investigadora URJC-Banco de Santander, 28922 Madrid, Spain; 6 Motion in Brains Research Group, Institute of Neuroscience and Movement Sciences (INCIMOV), Centro Superior de Estudios Universitarios La Salle, Universidad Autonoma de Madrid, 28049 Madrid, Spain; 7 La Paz Hospital Institute for Health Research, IdiPAZ, 28029 Madrid, Spain; 8 Department of Physiotherapy, Faculty of Sport Sciences, European University of Madrid, 28670 Villaviciosa de Odón, Spain; 9 Physiotherapy and Orofacial Pain Working Group, Sociedad Española de Disfunción Craneomandibular y Dolor Orofacial (SEDCYDO), 28009 Madrid, Spain; 10 Rehabilitation Service, La Paz Hospital, 28046 Madrid, Spain; veralgo83@gmail.com; 11 Centro de Salud Collado Villlalba (Estación), 28400 Madrid, Spain; rocio.conde@salud.madrid.org; 12 Faculty of Physiotherapy, Nursing and Podiatry, Complutense University of Madrid, 28040 Madrid, Spain; davidrodriguezsanz@ucm.es (D.R.-S.); cescalvo@ucm.es (C.C.-L.)

**Keywords:** low back pain, musculoskeletal diseases, neck pain, physical therapy

## Abstract

Background: Back pain is highly prevalent; in Spain, it produces a very high economic cost and the scientific evidence supporting treatments shows low to moderate evidence for exercise. Therefore, the aim of this study was to assess the effectiveness of a therapeutic group exercise protocol in reducing pain intensity and disability in patients with back pain in primary health care setting. Methods: A total sample of 149 patients who suffered from chronic non-specific back pain was selected. Patients received a therapeutic exercise protocol, including auto-mobilization exercises for the neck and lumbar regions, as well as core stabilization exercises. Pain intensity and disability were evaluated before and after the therapeutic exercise protocol. Results: Statistically significant differences (*p* < 0.05) were shown in pain intensity and disability for patients with non-specific neck and low-back pain, with an effect size from moderate to large. Conclusions: A therapeutic exercise protocol may provide beneficial effects upon disability and pain intensity in patients with chronic non-specific back pain, including neck and low-back pain conditions In addition, It could be considered for inclusion as a back-pain-approach program in primary healthcare.

## 1. Introduction

Currently, back pain presents a high lifetime prevalence from 77% to 85% and a great point prevalence from 30% to 43% [1,2]. Back pain, encompassing discomfort in the neck, thoracic, and lumbar regions, is a prevalent condition frequently encountered in physiotherapy practice [3].

In Spain (one of the countries that, together with Switzerland, has the largest number of prevalence studies for low-back and neck pain), non-specific neck and back pain showed a 1-year prevalence of 19.5 and 19.9%, respectively, and the cost of neck pain in primary care is quite high [4,5]. Low-back pain is the second most frequent reason to visit a physician for pain-relief in primary care [6]. Both chronic non-specific back and cervical pain conditions were more prevalent among women (26.4% for neck pain and 24.5% for back pain) than men (12.3% for neck pain and 15.1% for back pain) [7]. However, there is also evidence that there may be no association between sex and low-back pain, and that the sex differences found are due more to differences between populations at the cultural level or the characteristics of the health system [8]. Adults with an age range from 31 to 50 years were 1.5 times more likely to present non-specific low-back pain than adults with an age range from 16 to 30 years [9]. In addition, a strong association was reported between non-specific neck and low-back pain conditions [10,11]. 

Non-specific back pain may produce subsequent disability regarding daily living activities among patients who suffer from this musculoskeletal condition [12]. It seems that low-back pain with a recognized etiology (related to a particular pathology, such as infections, tumors, osteoporosis, fractures, structural deformities, inflammatory conditions, radicular syndrome, or cauda equina) occurs in only 10–15% of cases [13]. Conversely, low-back pain with an unidentified cause (non-specific low-back pain) account for 85–90% of all instances of low-back pain [13,14]. Indeed, some potential risk factors were described for disabling non-specific back pain, such as older age, female sex, the existence of back pain family history or a prior own history of back pain [15]. In addition, environmental agents such as low socioeconomic status and depression are present in the pathophysiology of non-specific low-back pain, and could be mediated in part by long-term reprogramming through epigenetic mechanisms [16]. Nevertheless, some factors such as spinal posture, illness factors and muscle endurance did not seem to be risk factors in order to develop disabling non-specific back pain [17]. However, a recent study found that age, sex, Body Mass Index (BMI), educational level, marital status, exercise frequency, history of low-back pain, work intensity, work posture, exposure to sources of vibration and psychological state were significantly associated with the occurrence of non-specific low-back pain [18].

In case of non-specific low-back pain, evidence guidelines recommend that patients should be active and receive good prognostic advice [19]. Physical inactivity is also recognized as a possible cause related to non-specific low-back pain [20]. Regarding interventions for non-specific back pain, low- to moderate-quality evidence for clinical improvements in pain intensity and disability has been reported, detailing some complementary health approaches (such as mindfulness, yoga, manual therapy or acupuncture), percutaneous electrical nerve stimulation, exercise, education or pharmacological interventions [21,22,23,24]. However, in a recent systematic review, manual therapy (Craniosacral Therapy) appears to be more effective than the other approaches that were compared (Yoga, Ayurvedic Massage, Pilates, Meditation, Meditation plus Yoga, Qigong, Tai Chi, and Dance) [25]. The available evidence supporting alternative treatments for non-specific neck pain, such as massage, acupuncture, manipulation, a soft cervical collar, electrotherapy, trigger point injections, botulinum injections, and yoga, as superior to sham or other conventional treatments, is not robust [26]. Thus, further interventional studies are necessary in order to find better interventions to generate clinical improvements in pain intensity and disability for patients who suffer from non-specific low back pain [23]. Indeed, exercise interventions, including some different physical activity modalities such as core stability and mobility exercises, may be considered as moderate-quality treatments to provide improvement in non-specific back pain disability for the mid-term and long-term in patients with chronic non-specific back pain [27,28]. Nevertheless, future research is needed to get beneficial guidance for a more rigorous study in this field [29,30].

According to these recommendations, we hypothesized that a therapeutic exercise protocol would show positive effects on the disability and pain intensity of patients who suffered from chronic non-specific back pain. Thus, the main aim of this study was to determine the effects of a therapeutic exercise protocol on disability and pain intensity in patients with chronic non-specific back pain in a hospital physical therapy unit. The secondary aim was to divide the sample according to median pain intensity and disability to assess post-intervention outcomes.

## 2. Materials and Methods

### 2.1. Study Design

Single-group retrospective-descriptive study with before–after design (Strengthening the Reporting of Observational studies in Epidemiology guidelines, STROBE) [31]. The study was approved by the Clinical Research Ethics Committee of the Puerta de Hierro Majadahonda Hospital, Madrid, Spain (act No 24-04-20, dated 24 April 2020). All the procedures were applied in accordance with the Helsinki Declaration. 

### 2.2. Participants

This study included patients treated at the Physiotherapy Outpatient Service of the Guadarrama Hospital (Madrid, Spain) between March 2015 and March 2017, as part of the program for the prevention of back pain focused on health education through the promotion of physical activity and the learning of therapeutic exercise guidelines carried out on patients referred from the Guadarrama Primary Health Center (Guadarrama, Spain), San Carlos Primary Health Center (San Lorenzo del Escorial, Spain) and El Escorial Primary Health Center (El Escorial, Spain); all belong to the Public Northwest Health Area of the Community of Madrid (Spain). The inclusion criteria established in the program were: (a) patients of both genders aged 20–80 years belonging to the primary care area, (b) presenting chronic non-specific neck or low-back pain (more than 3 months of evolution). Patients with (a) an initial score of less than 15 on the Neck Disability Index (NDI), (b) an initial score equal to or less than 20 on the Oswestry Lumbar Disability Scale (OS), (c) cancer diagnosis, decompensated hypertension, ventricular dysfunction, pregnancy, or any other alteration or disease with contraindications to the practice of exercise, or (d) acute spinal pathology or other associated musculoskeletal processes requiring individualized treatment were excluded.

### 2.3. Measurement Outcomes

Patients with chronic non-specific low-back pain were evaluated at the beginning and end of treatment using the Oswestry Lumbar Incapacity Scale (OS) [32] and the 10-point Numeric Pain Rating Scale (NPRS). Patients with chronic non-specific neck pain were evaluated at the beginning and end of the protocol using the NDI [33] and the 10-point NPRS [34].

The NDI is a self-administered scale consisting of 10 Likert-type questions that assess the impact of cervical pain in the activities of daily living (ADLs) on the domains of pain intensity, self-care, load-bearing, reading, headaches, concentration, work, driving, sleep and play activities. Each question is scored from 0 (no disability) to 5 (complete disability), with a maximum score of 50. Depending on this score, the scale presents no disability (<5), mild (5–14), moderate (15–24), severe (25–34) and complete disability (>34) levels. This is a scale with a standard error of measurement (SEM) of 4.3 [35], with excellent test-retest reliability (Intraclass Correlation Coefficient (ICC) = 0.96) [36] and consistency (Cronbach alpha = 0.92) [37]. It has a minimal clinically important change for patients with non-specific neck pain of 10 points [35].

The OS is also an administered scale of 10 Likert-type questions that evaluate functionality in relation to low-back pain in the dimensions of pain intensity, personal care, load-support, gait, seating, standing, sleep, sexual activity, social life and repercussions when traveling. Each question is scored from 0 (no disability) to 5 (complete disability), with a score expressed as a percentage, the maximum being 100%. Depending on this percentage, the scale presents minimum (<20%), moderate (20–40%), intense (40–60%), disability (60–80%) and maximum (>80%) levels. It has a SEM of 3.54 with excellent test-retest reliability (ICC = 0.97) and internal consistency (Cronbach alpha = 0.90) [33] It has a minimal clinically important change for patients with mechanically induced low-back pain of 12.8 points [38].

The NPRS 10 is a 10-point interval numeric scale that assesses perceived pain from 0 (no pain) to 10 (maximum pain imaginable). Depending on the score, chronic musculoskeletal pain can be categorized as mild (<6), moderate (6–7) and severe (>7) [39]. It has excellent test-retest reliability (Cronbach alpha = 0.98) [40] and a significant minimal clinical change for patients with described chronic pain of 1.7 points or a reduction of 28% [41].

### 2.4. Therapeutic Exercise Protocol

All patients were evaluated in a physiotherapy consultation at the beginning and at the end of treatment by an external evaluator who was blind to the treatments received by patients. The patients included in the program were assigned to one of the two groups, either the morning shift or the afternoon shift of the Physiotherapy Service of the Guadarrama Hospital. Each group of 8–10 patients was directed by one of the physiotherapists of the corresponding shift. Before the beginning of the program, the physiotherapists were trained in the administration of the therapeutic exercise protocol.

The patients carried out 10 sessions of about 30 min duration, over the course of 2 weeks, with a protocol of exercises of automobilization of the cervical and lumbar regions based on the McKenzie concept [42], as well as therapeutic exercise of the deep pre-vertebral musculature and activation of transverse and multifidus [43,44,45]. A more detailed description of this protocol is in the Supplementary Material (S1: Detailed description of the therapeutic exercise protocol).

### 2.5. Statistical Analysis

For statistical analysis, the study used program R Ver. 3.3.3. (R Foundation for Statistical Computing, Institute for Statistics and Mathematics, Welthandelsplatz 1, 1020 Vienna, Austria). The level of significance was established at *p* < 0.05. The Kolmogorov– Smirnoff test with Lillieford correction was used to determine the non-normal distribution of baseline and outcome variables. Qualitative variables were described in absolute values and frequencies and quantitative variables with mean and standard deviation (SD), or with median and interquartile range (IQR), depending on whether or not there was a normal distribution. 

The Wilcoxon Signed Rank test was used on the initial and final scores of the Oswestry, NDI and NPRS 10 lumbar and cervical scales. Among the categorized outcome variables, the McNemar–Bowker test was applied with post hoc tests with Bonferroni correction. The Mann–Whitney or Kruskal–Wallis U test was applied between the result and basal variables. On the other hand, the exact Fisher test was applied between the categorized result variables and the categorical basals. On the other hand, the Mann-Whitney U test or the Kruskal-Wallis test was applied to the quantitative variables, and the Fihser exact test to the qualitative variables, between the post-treatment outcome variables and the pre-treatment variables (NPRS10, NDI and Oswestry) dichotomized based on their median.

The effect size between the quantitative variables (r) was defined as <0.20 (not relevant), ≥0.20 and <0.50 (small), ≥0.50 and <0.80 (moderate) and ≥0.80 (large); in the qualitative variables the effect size (Cohen g) was defined as ≥0.05 and <0.15 (small), ≥0.15 and <0.25 (moderate) and ≥0.25 (large) and (Cramer V) as <0.212 (small), ≥0.212 and <0.354 (medium) and ≥0.354 (large).

## 3. Results

A total of 149 patients were treated, 32 men and 117 women with a mean age of 60 (50, 69) years, the median age of majority (56.4%). Almost half (46.3%) had chronic non-specific low-back pain and did not report dorsal pain (84.6%) (Table 1).

### 3.1. Chronic Non-Specific Low-Back Pain Patients

The Mann–Whitney U test (Kruskal–Wallis in the case of categorized age) does not indicate significant differences between the initial and final outcome variables as a function of the basal variables except for the uncategorized age (*p* > 0.001), although the presence of basal differences does not allow conclusions to be drawn.

Fisher’s exact test of the initial and final categorized outcome variables, as a function of the categorized baseline variables, does not indicate significant differences (*p* > 0.05) except in the case of NPRS 10, although the presence of baseline differences does not allow conclusions to be drawn.

The Wilcoxon Signed Rank test of uncategorized initial versus final outcome variables indicates significant differences: (a) initial vs final OS scale (Z = 8.348, *p* < 0.001) with a difference of 8 [12 (median) 95% CI (9, 13)] points and a moderate and significant effect size [r = 0.759, 95% CI (0.651, 0.837)], (b) NPRS 10 initial lumbar vs. final (Z = 8.046, *p* < 0.001) with a median difference of 2 [95% CI (2, 3)] points and a moderate and significant effect size [r = 0.731, 95% CI (0.622, 0.812)].

**Figure 1:**
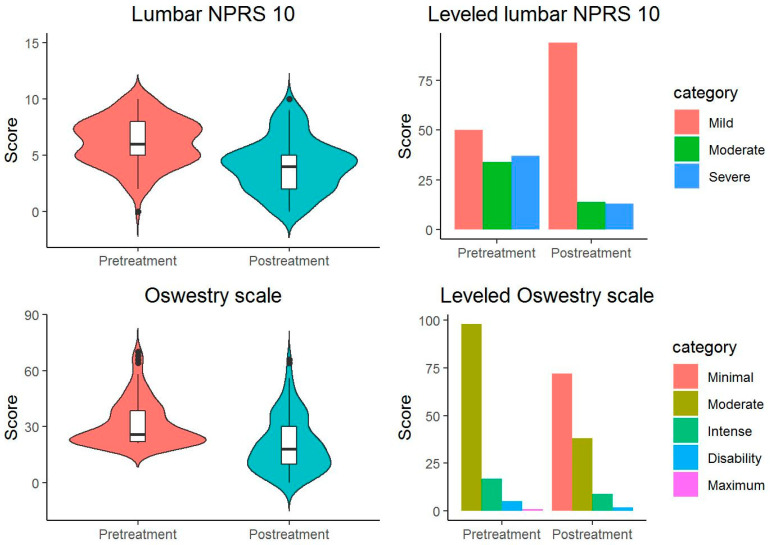
Starting and ending scores on the Oswestry scale and NPRS 10 lumbar (black dots: outliers data points).

The McNemar–Bowker test of the initial and final categorized outcome variables shows significant differences: (a) in the proportion of the OS scale categories (X^2^(3) = 9.333, *p* = 0.025) with a large overall effect size [Cohen’s g = 0.375 95% CI (0.167, 0.5)], specifically, between the Intense and Moderate categories (*p* = 0.038) with a large effect size [Cohen’s g = 0.333 95% CI (0.1, 0.5)], (b) in the proportion of the NPRS 10 scale categories categorized (X^2^(3) = 38.23, *p* < 0.001) with a large overall effect size [Cohen’s g = 0.411 95% CI (0.336, 0.481)], among the categories (a) Slight-Moderate (*p* < 0.001) with a large effect size [Cohen’s g = 0.423 95%. CI (0.3, 0.5)], and (b) Slight-Severe (*p* < 0.001) with a large effect size [Cohen’s g = 0.423 95% CI (0.3, 0.5)] (Figure 1 and Table 2).

### 3.2. Patients with Chronic Non-Specific Neck Pain

The Mann–Whitney U test (Kruskal–Wallis in the case of categorized age) does not indicate significant differences in the initial and final outcome variables as a function of the basal variables, except for uncategorized age (*p* > 0.001) although the presence of basal differences does not allow conclusions to be drawn.

Fisher’s exact test of the initial and final categorized outcome variables as a function of the categorized baseline variables does not indicate significant differences (*p* > 0.05), except in the case of the final score in NPRS 10 as a function of the presence of dorsal pain (*p* = 0.028) with a medium effect size (Cramer’s V = 0.29, IC 95% [0, 1]). The post hoc test fails to detect between which levels significant changes occur; however, it is evident that more than half of the patients (89.1%) presented at the end of treatment a mild level of cervical pain without being associated with the presence of dorsal pain.

Signal testing of the Wilcoxon ranges of uncategorized initial versus final outcome variables indicates significant differences (Table 3): (a) initial vs. final NDI (Z = 5.887, *p* < 0.001) with a difference of 11 [8 (median) 95% CI (6, 11)] points and a large and significant effect size [r = 0.658, 95% CI (0.52, 0.779)], (b) NPRS 10 cervical initial vs. final (Z = 6.803, *p* < 0.001) with a difference of 3 [95% CI (2, 3)] points and a moderate and significant effect size [r = 0.761, 95% CI (0.641, 0.842)].

**Figure 2:**
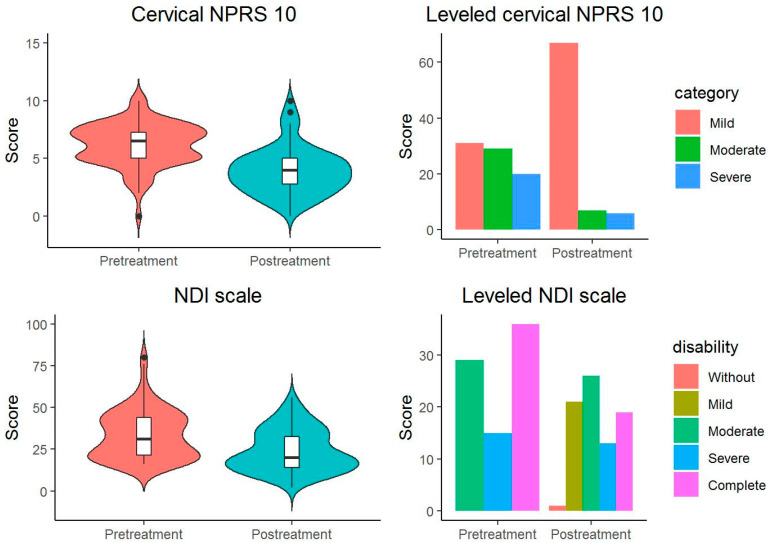
Starting and ending scores on the NDI and NPRS 10 cervical scales (black dots: outliers data points).

The McNemar–Bowker test of the initial and final categorized outcome variables shows significant differences: (a) in the proportion of categories on the NDI scale (X^2^ (3) = 11,807, *p* = 0.008) with a large overall effect size [Cohen’s g = 0.293 95% CI (0.18, 0.433)], specifically between the categories Moderate Disability and Complete Disability (*p* = 0.01) with a large and significant effect size [Cohen’s g = 0.423 95% CI (0.25, 0.5)]; (b) in the proportion of the categories of the categorized NPRS 10 scale (X^2^(3) = 39, *p* < 0.001) with a large overall effect size (Cohen’s g = 0.5), between the categories (a) Mild-Moderate (*p* < 0.001) with a large effect size (Cohen’s g = 0.5), and (b) Mild-Severe (*p* < 0.001) with a large effect size (Cohen’s g = 0.5) (Figure 2 and Table 3).

### 3.3. Median Pain Intensity Between-Group Comparison

The chronic non-specific low-back and neck pain populations were split according to median (Me) pain intensity to see the post-intervention behavior of the self-reported disability variable and vice versa.

### 3.4. Patients with Chronic Non-Specific Low-Back Pain

The pre-intervention mean of NPRS 10 for patients with upper pain intensity was 8.68 ± 5.30, while the pre-intervention mean of NPRS 10 for patients with lower pain intensity was 4.48 ± 1.28. When comparing the mean values of the OS variable between these groups at post-intervention, statistically significant between-groups differences were found with a small effect size (mean differences [MD] = −10.785 (−15.761, −5.81); *p* < 0.001, *r* = 0.37). In addition, the pre-intervention mean of OS for patients with upper disability was 42.24 ± 13.37, while the pre-intervention mean of OS for patients with lower disability was 22.48 ± 1.88. When comparing the mean values of the NPRS 10 variable between these groups at post-intervention, statistically significant between-groups differences were found with a small effect size (MD = −0.997 (−1.823, −0.171); *p* = 0.008, *r* = 0.242).

### 3.5. Patients with Chronic Non-Specific Neck Pain

The pre-intervention mean of NPRS 10 for patients with upper pain intensity was 7.67 ± 0.86, while the pre-intervention mean of NPRS 10 for patients with lower pain intensity was 4.72 ± 1.26. When comparing the mean values of the NDI variable between these groups post-intervention, statistically significant between-groups differences were found with a small effect size (MD = −8.1 (−14.048, −2.152); *p* = 0.01, *r* = 0.285). However, when comparing the mean values of the NPRS 10 variable between groups with upper (45.45 ± 10.40) and lower (21.50 ± 4.65) NDI, no statistically significant differences were found between the groups (MD = −0.675 (−1.606, 0.256); *p* = 0.2).

## 4. Discussion

This study aimed to evaluate the effectiveness of a therapeutic group exercise protocol for non-specific back pain. After our program, the pain intensity reduced significantly. Our results also showed that this exercise program was acceptable, and participants improved their back health status [46].

A recent network meta-analysis [47] concluded that no single type of exercise is the best treatment for patients with low back pain. However, that study showed that active therapies, such as Pilates, aerobic exercise, stabilization/motor control exercise, and resistance exercise, are more effective in improving pain and disability. This network meta-analysis concluded that the three likely best exercises obtained better outcomes for improve pain than McKenzie in 16 points on pain outcomes. Moreover, Pilates, yoga, aerobics, stabilization/motor control exercise and water-based exercise showed an improvement of 11 points on the Oswestry disability index that is considered clinically relevant [48], but not for the McKenzie exercise, which only scored 3 points; but, in contrast, in our study we found 8 points of difference. Although 11 points are considered clinically relevant and in our study we only reached 8 points, the range established for minimally detectable changes in the Oswestry disability index is between 4–16 points, thus our study achieved an acceptable range of improvement [48]. 

However, there is low-quality evidence in the literature that McKenzie was not effective for pain and function in patients with chronic low-back pain, and a low quality of evidence that Pilates, stabilization/motor control, resistance and aerobic exercise were able to improve pain and function [47]. 

Several studies where they applied McKenzie exercises found an improvement between 50% to 68,76% in non-specific low-back pain intensity, but short-term and not for the long term [49,50,51]; these outcomes are superior to the results obtained in our study, where the percentage of change of improvement reached 33.33%. 

The percentage of change of improvement for disability in our study reached 30.7%; this outcome is lower than previous studies, where they reached between 50% to 77.77% from immediately to 3 months after [49,51,52], but in a recent study no differences in disability were found for McKenzie exercises [50]. Two previous observational studies without a control group have shown that the McKenzie method allows for the classification of patients with neck pain and provides improvements in patients in terms of neck disability [53,54]. In a recent published systematic review with meta-analysis, they concluded that McKenzie provides very small but statistically significant improvements in neck pain of all severity compared to control interventions [55]. However, a recent Cochrane review of the McKenzie method for treating non-specific (sub)acute low-back pain found by testing, with low to very low certainty, that the treatment-effects observed with respect to pain and disability were not clinically significant [56].

Other therapeutic exercises and activities, such as Pilates, neuromuscular balls or yoga, are often recommended for general back pain. Researchers have conducted some studies to improve the scientific evidence on core stability, as shown in recent meta- analyses that demonstrate that core stability exercises are highly recommended for improving physical function and reducing pain in chronic low-back patients in the short term [30,57,58].

Therapeutic exercise is the most effective intervention for the management of back pain [59]. Physical activity and therapeutic exercise lead to an activation of endogenous pain-inhibitory mechanisms, and also lead to a reduction in sensitivity to noxious stimuli related to the modality of physical activity [60,61]. This is perhaps the main mechanism that explains how the pain score could be alleviated and how the NDI/OS improved with the therapeutic multimodal exercise protocol used in the present study, in addition to the fact that it has been widely demonstrated that therapeutic exercise is able to promote bone formation, stimulating the metabolism and bone remodeling through mechanical loading (compression, tension and tissue shear), improving hormonal regulation (estrogens, parathyroid hormone, testosterone and glucocorticoids), facilitating the regulation of signaling pathways and the stimulation of angiogenic–osteogenic responses [62,63].

Hayden et al. [64] show how resistance exercises, which improve the abdominal wall muscles and back muscles, are effective in the treatment of low-back pain. This finding is consistent with other research which reports that psychotherapy (relaxation techniques) is effective in the treatment of employees with chronic low-back pain [65].

Another study offers inconclusive evidence for the effectiveness of the non-invasive management of cervicobrachial pain, showing that a manual therapy intervention combined with exercise resulted in a better health status in patients than manual therapy [66]. In addition, a treatment intervention for non-specific neck pain adding core stability exercises provided more reliable evidence for future studies [67]. However, in contrast to Cochrane’s systematic reviews of the effectiveness of exercise on neck pain, they report that strengthening and resistance/stabilization exercises have a small to large impact on neck pain [68,69]. It has even been found that the effect of combining exercise with other therapies such as manual therapy increases positive results in non-specific neck pain and disability [70,71].

In a recent clinical trial involving 45 patients experiencing chronic neck pain [72], the participants were divided into three groups: Group 1 received conventional treatment, Group 2 received conventional treatment along with deep cervical flexor-training, and Group 3 received conventional treatment combined with neck-and-core-stabilization exercises. The study revealed that, in addition to conventional treatment, implementing trunk-stabilization exercises or deep cervical flexor-training for patients with chronic neck pain may yield a greater effectiveness in reducing pain and disability, while increasing the range of motion, compared to conventional treatments alone.

This study shows that, after a two-week intervention, therapeutic exercise improved pain intensity, functional disability, and patient satisfaction, as well as the activation of transversus of abdominis and superficial fibers of multifidus muscles in subjects with chronic non-specific low-back pain [73].

Some studies offer low–moderate quality evidence that control-stabilization exercise interventions improve pain intensity and disability in subjects with chronic non-specific low-back pain, compared to a passive control group or to other exercises [74].

Our study shows a pain-status improvement in patients with chronic non-specific back pain who had completed a therapeutic exercise protocol. Exercise therapy by itself, related with relaxation, is a therapeutic option commonly prescribed in the treatment of patients with chronic non-specific back pain. It is considered as an interesting intervention for increasing the range of movement [75]. Improvement in range of movement is related to increased blood flow to muscles and reduced stiff joints [76]. Our results are in line with the conclusions of a recent systematic review, which found that non-pharmacological treatments present strong evidence for chronic musculoskeletal pain-relief in primary care, and with a recent umbrella review with meta-meta-analysis that found orthopedic manual therapy improved endogenous analgesia [77,78].

### Limitations

The main limitation of the present study lies in the absence of a comparator group, due to its character as a habitual clinical practice, so the results have to be interpreted with caution.

In addition, patients’ expectations were not evaluated. In this context, Ballestra et al. [79] demonstrated that specific patient expectations, such as anticipating a personalized treatment with regular follow-ups, hoping for optimal outcomes, having realistic or accepting attitudes towards alleviating health issues, fostering effective dialogue and communication, seeking individual recognition, and desiring comprehensive explanations of their condition, may be associated with improved recovery outcomes.

A recent systematic review found evidence of increased levels of the proinflammatory biomarkers CRP, IL-6 and TNF-α and decreased levels of the anti-inflammatory biomarker IL-10 in patients with acute and chronic non-specific low-back pain [80]. Therefore, we believe that it would have been valuable to consider incorporating a laboratory blood study into the analysis.

## 5. Conclusions

A therapeutic exercise protocol may provide beneficial effects upon disability and pain intensity in patients with chronic non-specific back pain, including non-specific neck and low-back pain conditions. It could be considered for inclusion as a back-pain-approach program in primary healthcare. Further studies are necessary, including a control group, in order to compare this therapeutic exercise protocol in patients who suffer from back pain.

## Figures and Tables

**Table 1:** Baseline Demographic characteristics of the patients.

n		149
Turn	Afternoon	58 (38.9%)
	Morning	91 (61.1%)
Age		60 [50, 69]
Leveled age	Young	17 (11.4%)
	Middle age	84 (56.4%)
	Older	48 (32.2%)
Gender	Female	117 (78.5%)
	Male	32 (21.5%)
Non-specific neck pain/Low back pain	Non-specific neck pain	28 (18.8%)
	Non-specific low back	69 (46.3%)
	Non-specific neck and low back pain	52 (34.9%)
Non-specific dorsal pain	No	126 (84.6%)
	Yes	23 (15.4%)

Data expressed with median and interquartile range [IQR] or with absolute and relative values (%).

**Table 2:** Outcome variables in patients with chronic non-specific low back pain.

		Initial	Final	Difference (Median 95% CI)	*p*	r (95% CI)
Oswestry		26 [22, 40]	18 [10, 30]	8 (12 [9, 13])	<0.001 ^a^	0.759, (0.651, 0.837)
						Cohen’s g (95% CI)
					0.025 ^a^	0.375 (0.167, 0.5)
	Minimum	-	72 (59.5%)			
	Moderate	98 (81%)	38 (31.4%)		0.038 ^a^	0.333 (0.1, 0.5)
	Intense	17 (14%)	9 (7.4%)		0.038 ^a^	0.333 (0.1, 0.5)
	Disability	5 (4.1%)	2 (1.7%)			
	Maximum	1 (0.8%)	-			
						**r (95% CI)**
NPRS 10		6 [5, 8]	4 [2, 5]	2 [2 (2, 3)]	<0.001 ^a^	0.731 (0.622, 0.812)
						Cohen´s g (95% IC)
					<0.001 ^a^	0.411 (0.336, 0.481)
	Mild	50 (41.3%)	94 (77.7%)		<0.001 ^a^	0.423 (0.3, 0.5),
	Moderate	34 (28.1%)	14 (11.6%)		<0.001 ^a^	0.423 (0.3, 0.5),
	Severe	37 (30.6%)	13 (10.7%)		<0.001 ^a^	0.423 (0.3, 0.5).

Oswestry: Oswestry Lumbar Disability Scale; NPRS 10: 10 Point Numerical Pain Scale. Data expressed with median and interquartile range [IQR] or with absolute and relative values (%). 95% CI: 95% confidence interval. ^a^ Significant if *p* < 0.05.

**Table 3:** Outcome variables in patients with chronic non-specific neck pain.

		Initial	Final	Difference (Median 95% CI)]	*p*	r (95% CI)
NDI		31 [21.5, 44]	20 [14, 32.5]	11 [8 (6, 11)]	<0.001 ^a^	0.658 (0.52, 0.779)
						Cohen´s g (95% CI)
					0.008 ^a^	0.293 (0.18, 0.433)]
	No disability	-	1 (1.2%)			
	Mild	-	21 (26.2%)			
	Moderate	29 (36.2%)	26 (32.5%)		0.01 ^a^	0.423 (0.25, 0.5)
	Severe	15 (18.8%)	13 (16.2%)			
	Complete disability	36 (45%)	19 (23.8%)		0.01 ^a^	0.423 (0.25, 0.5)
						**r (95% CI)**
NPRS 10		6.5 [5, 7.75]	4 [3, 5]	2.5 [3 (2, 3)]	<0.001	0.761 (0.641, 0.842)
						Cohen´s g
					<0.001 ^a^	0.5
	Mild	31 (38.8%)	65 (81.2%)		<0.001 ^a^	0.5
	Moderate	27 (33.8%)	7 (8.8%)		<0.001 ^a^	0.5
	Severe	20 (25%)	6 (7.5%)		<0.001 ^a^	0.5

NDI: Cervical Disability Index; NPRS 10: 10 Point Numerical Pain Scale. Data expressed with median and interquartile range [IQR] or with absolute and relative values (%). 95% CI: 95% confidence interval. ^a^ Significant if *p* < 0.05.

## Data Availability

The data presented in this study are available upon request from the corresponding author. The data are not publicly available due to ethical restrictions.

## Supplementary Materials

The following supporting information can be downloaded at: https://www.mdpi.com/article/10.3390/jcm12206478/s1, detailed description of the therapeutic exercise protocol.
