# Supplementary material for: Effects of a Therapeutic Exercise Protocol for Patients with Chronic Non-Specific Back Pain in Primary Health Care: A Single-Group Retrospective Cohort Study
Source: J Clin Med. 2023 Oct 12;12(20):6478. doi: 10.3390/jcm12206478 (PMC10607108; doi:10.3390/jcm12206478)
Supplement: Supplementary file 1 [file jcm-12-06478-s001.zip › jcm-2589346-supplementary.pdf]

Between exercise and exercise ask for a soft breath, inhaling through the nose and exhaling through the mouth.

SUPINE POSITION:

Take air in through your nose and release it through your mouth slowly; keep one hand on your chest and the other on your gut.

1. Take a breath noticing as if you were inflating a ball in your chest, while your hand in your gut does not move.

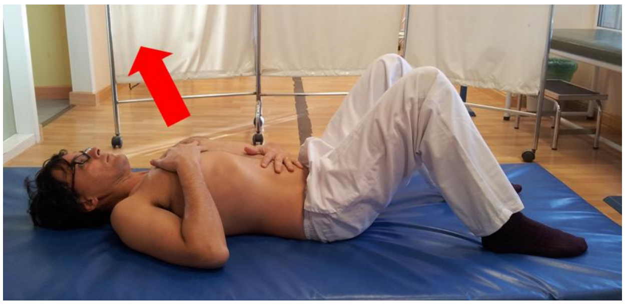

2. Take a breath noticing as if you were inflating a ball in your gut while the one in your chest is not moving.

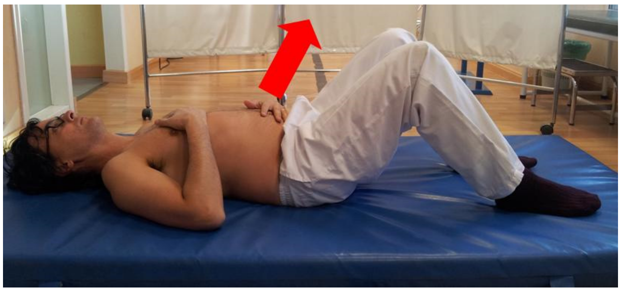

3. Now, fill the chest ball and, without releasing the air, pass it to the gut ball; again pass it to the ball of the chest and release the air.

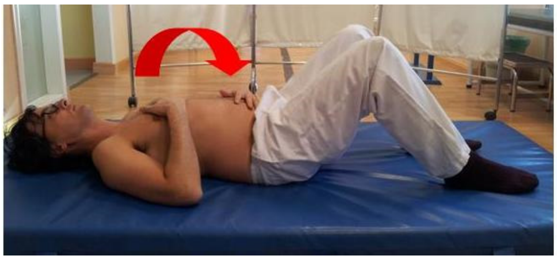

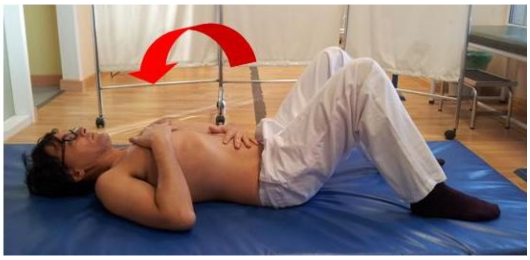

Total time for the three exercises, 10 minutes, each exercise is repeated 5 times.

4. Put one or two hands on the chin and, helping yourself with these, bring the chin towards the neck, pulling out "dewlap" and noticing how the back of the neck tightens; hold the position for a count of 5 and relax.

Pº Molino del Rey, 2
Tlfn .: 91 856 20 00/91 856 20 02
Fax: 91 856 18 99
Email: enfermerí a.hgua@salud.madrid.org
28440 Guadarrama - Madrid

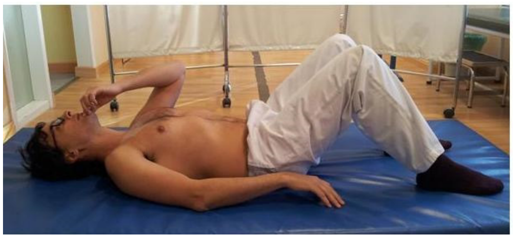
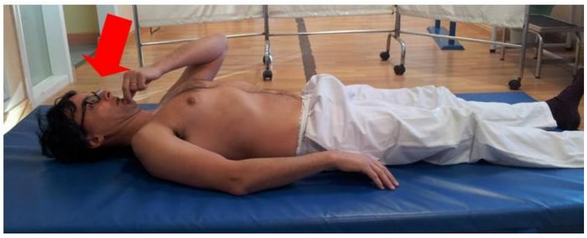

5 repetitions.

5. Now that the head is resting heavily on the pillow, turn to the right until you feel tension in the left part of the neck and keep counting to 5; repeat to the left.

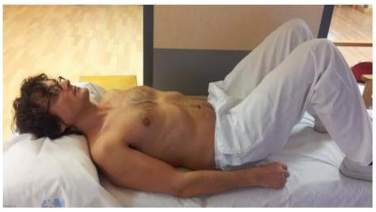
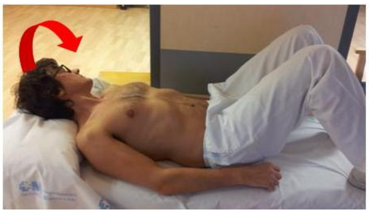
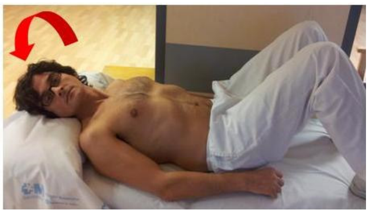

3 alternate repetitions left and right.

6 . Bend one leg and then the other; With both hands bring your knees to your chest and pull them until you feel the back of your back tense; hold the position for a count of 5 and return to the starting position by lowering one leg and then the other.

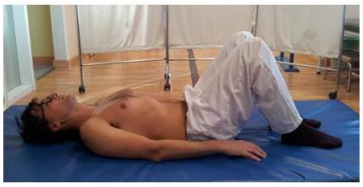
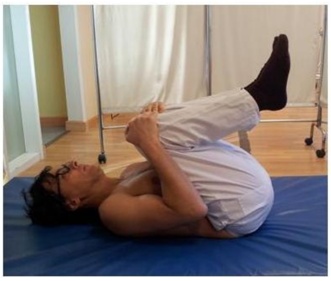
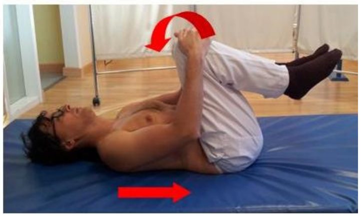

5 repetitions.

7. Separate the arms from the body and rotate the knees to the left until you feel tension in the left side; count to 5; repeat right.

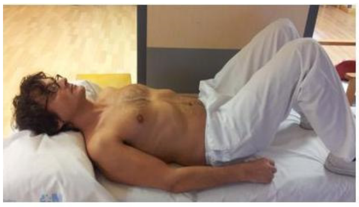
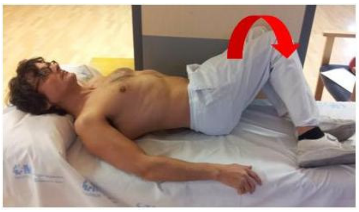
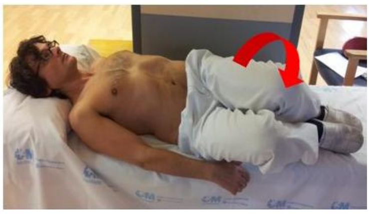

3 alternate repetitions left and right.

8. Now push your feet against the floor and squeeze your buttocks so that your pelvis is slightly off the floor; avoid tightening the back of the thighs and arching the back; count to 5.

Pº Molino del Rey, 2
Tlfn .: 91 856 20 00/91 856 20 02
Fax: 91 856 18 99
Email: enfermerí a.hgua@salud.madrid.org
28440 Guadarrama - Madrid

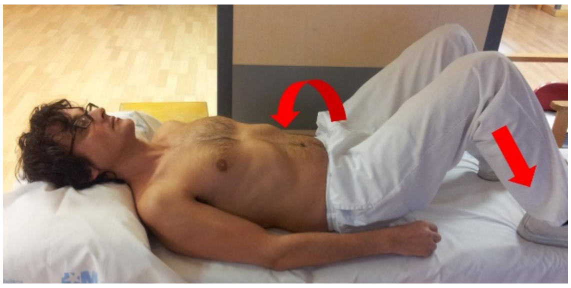

5 repetitions.

9. Put your hands crossed on your chest and take your shoulders and head off the ground a little, looking at the ceiling, tensing your abs; count to 5.

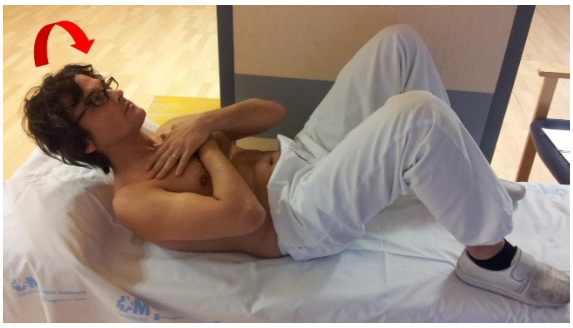

5 repetitions.

10. Keep one hand on your chest and the other on your stomach while you breathe in through your nose and release them, as slowly as possible, through your open mouth; each time you release the air, put the gut in as if you wanted to bring your navel against the table; try with your hand to keep it from falling while doing it.

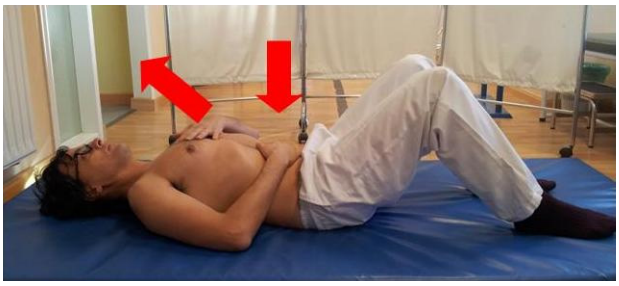

5 repetitions.

QUADRUPEDIA:

Between each exercise it is allowed to move the legs and arms to discharge them.

11. Stay on all fours with your back in a comfortable, neutral position, neither too arched nor too flexed; raise one leg backwards taking off the foot of the mat and raising it as horizontal as possible, holding for 1 sg, and lower; try not to move your back while doing the exercise.

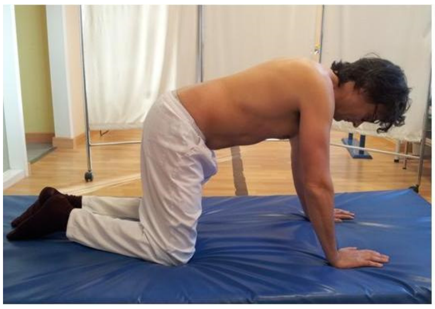
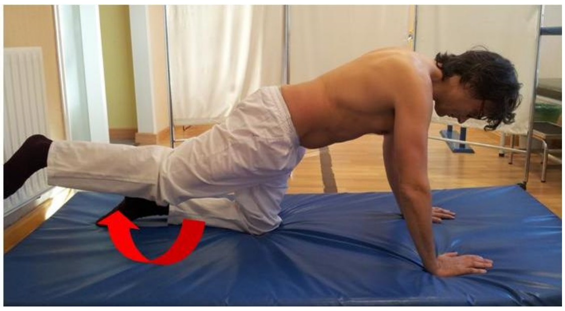

3 repetitions with each leg.

12. Now arch your back by "humping" and counting to 5, and then "sink it by stretching", also counting to 5.

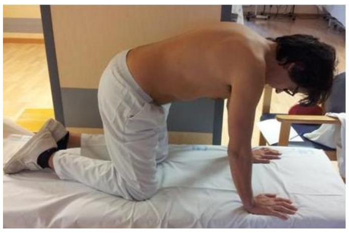
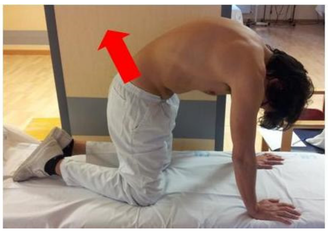
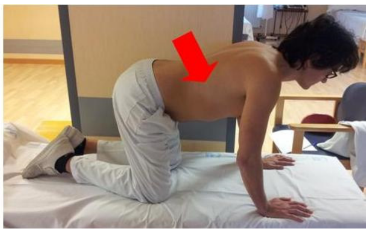

5 repetitions.

13. In the same position described above, raise one arm as close to the horizontal position as possible, holding the position for 1 sg and lower; try not to move your back while doing the exercise.

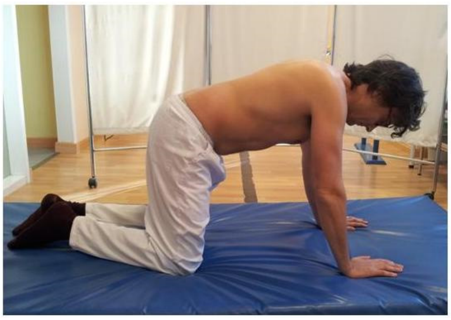
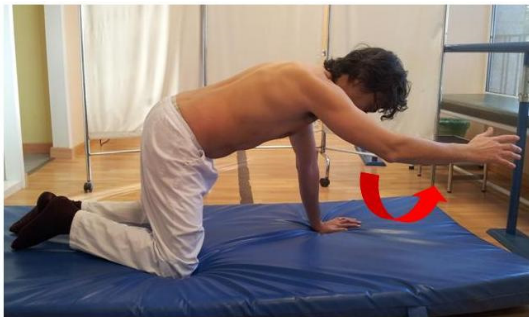

3 repetitions with each arm.

14. Sit on your heels taut your sides and count to 5.

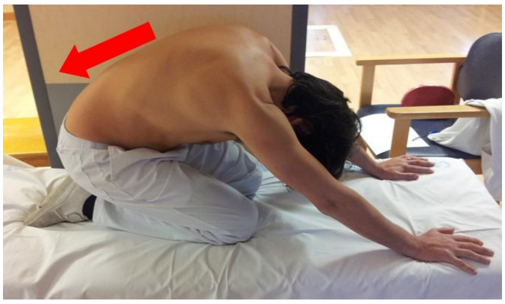

Pº Molino del Rey, 2
Tlfn .: 91 856 20 00/91 856 20 02
Fax: 91 856 18 99
Email: enfermerí a.hgua@salud.madrid.org
28440 Guadarrama - Madrid

5 repetitions.

SITTING:

Sit in a chair with your back against the back.

15. Tilt your head to one side, place your hand on the side it tilts around the opposite part of the head; pull with your hand until you feel tension on the side of the neck; hold the position for a count of 3 and relax.

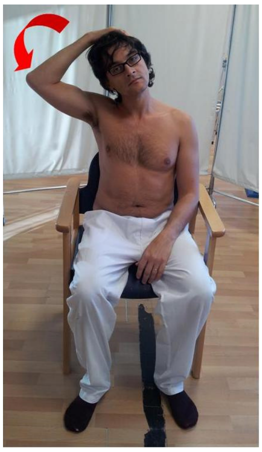

3 alternate repetitions left and right.

16. Direct your gaze towards the floor, rest both hands on the nape of the neck with your elbows together, and pull them towards the floor until you feel tension in the back of the neck; hold the position for a count of 3 and relax.

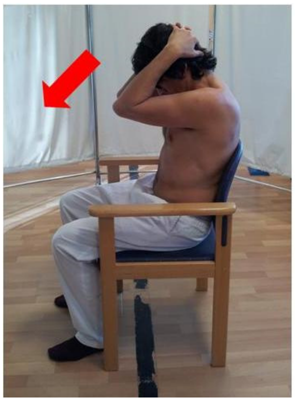

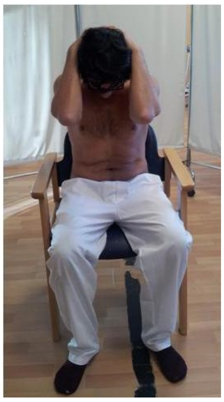

5 repetitions.

17. Put your hands behind your head and turn the trunk first to the left, counting to 5, and then to the right, counting to 5 again.

Pº Molino del Rey, 2
Tlfn .: 91 856 20 00/91 856 20 02
Fax: 91 856 18 99
Email: enfermerí a.hgua@salud.madrid.org
28440 Guadarrama - Madrid

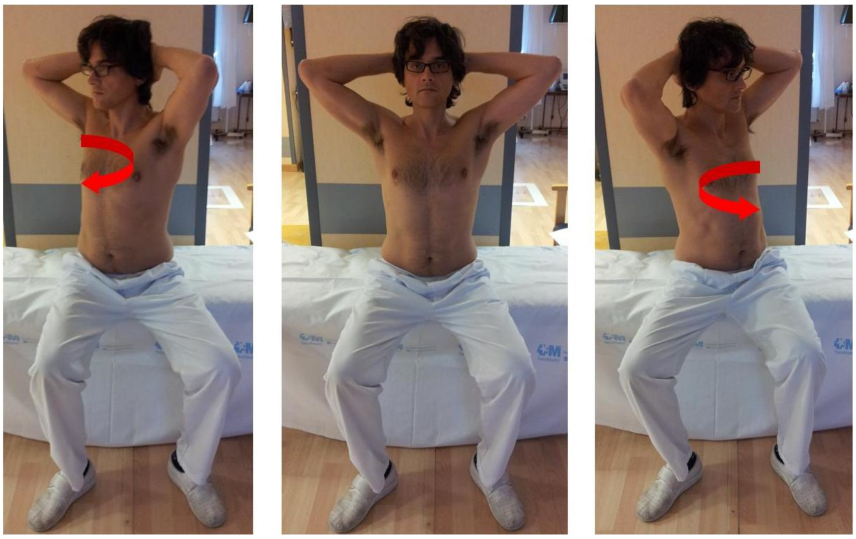

3 alternate repetitions left and right.

18. Bend your waist forward until you touch the ground with your hands; slide them back as far as you can by touching the floor with the back of your hands; hold the position for a count of 3, then return to the starting position and repeat, but now grab your ankles with your hands and pull your body down with them holding the position again for a count of 3.

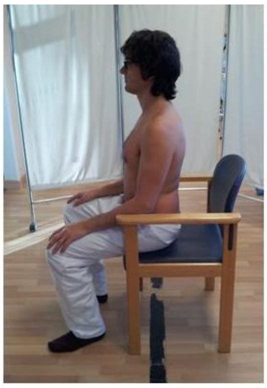

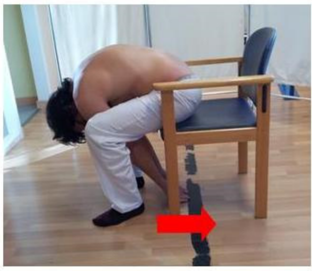

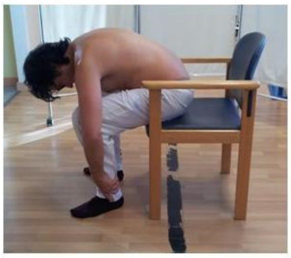

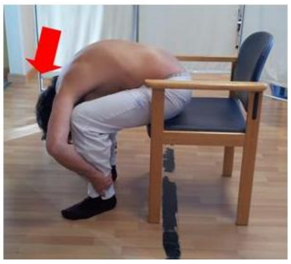

3 repetitions.

STANDING:

19. Support both hands on the lower part of the back, surrounding the waist; tilt your body back as far as you can while pushing forward with your hands; it is important to keep the knees straight; hold the position for a count of 3 and relax.

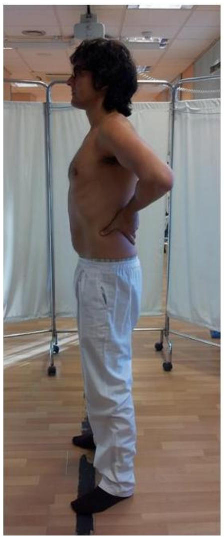

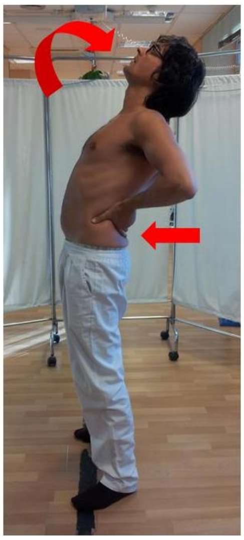

5 repetitions.

Pº Molino del Rey, 2
Tlfn .: 91 856 20 00/91 856 20 02
Fax: 91 856 18 99
Email: enfermerí a.hgua@salud.madrid.org
28440 Guadarrama - Madrid
